# Report on Morbus Coxarius

**Published:** 1861

**Authors:** 


Report on Morbus Coxarius; or Hip Disease.—By Lewis A. Sayre, M. D., Resident Physician of the City of New York; Surgeon to Bellevue Hospital, &c., &c.

This is a monograph of 94 pages, reprinted from the Transactions of the American Medical Association for 1860. It is the Report that attracted more attention, and is of more practical value than any other presented to the Association during its last annual meeting. After a brief description of the anatomy of the hip-joint, Dr. Sayre discusses the causes and pathology of hip disease, gives a detailed account of the symptoms in the several stages, accompanied by cuts for illustration; giving the treatment adapted to each stage of the disease, with a full account of the mechanical appliances necessary, and concludes with a summary of the cases thus far treated, with the results. To give any adequate idea of the author's views and method of treating the disease, would compel us to copy the greater part of the report itself. Its publication makes an era in the history and treatment of hip disease, and we advise every reader to obtain a copy of it, together with much other valuable matter, by sending *three* dollars to Dr. Casper Wistar, of Philadelphia, for the volume of Transactions of the American Medical Association, for 1860.

